# What is ethosome photothermal therapy?

**DOI:** 10.1111/srt.13799

**Published:** 2024-06-09

**Authors:** Kyung‐Tae Bae, Kyu‐Ho Yi

**Affiliations:** ^1^ It's Me Clinic Sejong South Korea; ^2^ Division in Anatomy and Developmental Biology Department of Oral Biology Human Identification Research Institute BK21 FOUR Project Yonsei University College of Dentistry 50–1 Yonsei‐ro Seodaemun‐gu Seoul South Korea; ^3^ Maylin Clinic (Apgujeong) Seoul South Korea

Dear Editor

Photodynamic therapy (PDT) has been a popular method for acne treatment and continues to be widely used in many clinics. However, PDT faces limitations due to the prolonged incubation period required and the various side effects associated with photosensitizers, such as erythema, hyperpigmentation, and dermatitis. An innovative breakthrough was achieved by applying gold PTT (photothermal therapy), originally used in cancer treatment, to the destruction of sebaceous glands, thus overcoming some of the limitations of PDT. Nevertheless, a significant challenge remains as natural penetration without mechanical assistance is difficult.^1^

To address this, gold PTT using ethosomes has been developed, representing a new generation of PTT that surpasses previous limitations and demonstrates its efficacy. Ethosomes, advanced lipid‐based nanosystems, consist of phospholipids and ethanol. Ethanol increases lipid layer fluidity and loosens the skin barrier, facilitating efficient penetration. This allows the phospholipids in ethosomes to naturally merge with the skin's lipid layers, enhancing the penetration of their contents quickly and effectively.^2^, ^3^, ^4^

The dermis is derived from two distinct lineages. Fibroblasts from Lineage^2^ form the papillary dermis and dermal papillae, playing a role in the growth and regulation of hair follicles and hair shafts. In contrast, fibroblasts from Lineage^3^ contribute to the formation of the reticular dermis and hypodermis. While the papillary dermis has the ability to regenerate after damage, the reticular dermis, when damaged, results in scarring. The sebaceous glands we aim to treat are located near hair follicles, where the surrounding cells can regenerate after damage. Therefore, PTT using hyperthermia can selectively destroy the sebaceous glands without the risk of scarring.^5^, ^6^

As aging progresses, the skin experiences a decrease in collagen and elastin, along with changes in the ratio of collagen types 1 and 3. Additionally, the structure of the basement membrane between the epidermis and dermis gradually weakens. To strengthen this, stimulation of collagen regeneration is necessary, and PTT can be an effective method for achieving this through hyperthermia.^7^ Ethosome PTT consists of five layers, structured as ethosome‐gold NP‐platinum NP‐ethosome‐active ingredient. The outermost layer of ethosome alone exhibits excellent penetration capabilities. However, the use of penetration devices such as microneedle therapy systems, plasma, or ultrasound can enhance the depth of penetration, thereby enabling more effective treatment.^8^

When ethosome PTT penetrates the skin, exposure to a light source causes the gold and platinum nanoparticles to generate heat through the surface plasmon resonance (SPR) effect. SPR occurs due to the collective oscillation of free electrons on the metal surface when they absorb light of a specific wavelength, resulting in heat generation. This effect is influenced by the material, size, thickness, and shape of the SPR metal, which determine the absorbed wavelength. Additionally, the thermal effect varies based on the laser's wavelength, power, spot size, exposure time, mode (continuous or pulsed), and the properties of the target tissue. Excessive energy exposure can cause the metal nanoparticles to fragment, affecting heat efficiency. The optimal laser for use after ethosome PTT penetration remains a subject of debate. Typically, a 1064 nm wavelength laser is used for acne treatment, while Q1064 or P1064 lasers are commonly used for pigment treatment.^9^, ^10^, ^11^ A 16‐year‐old patient came in had chief complaints of acne and had three treatment sessions with ethosome PTT with Q1064 laser combination therapy (Figure 1).

**FIGURE 1:**
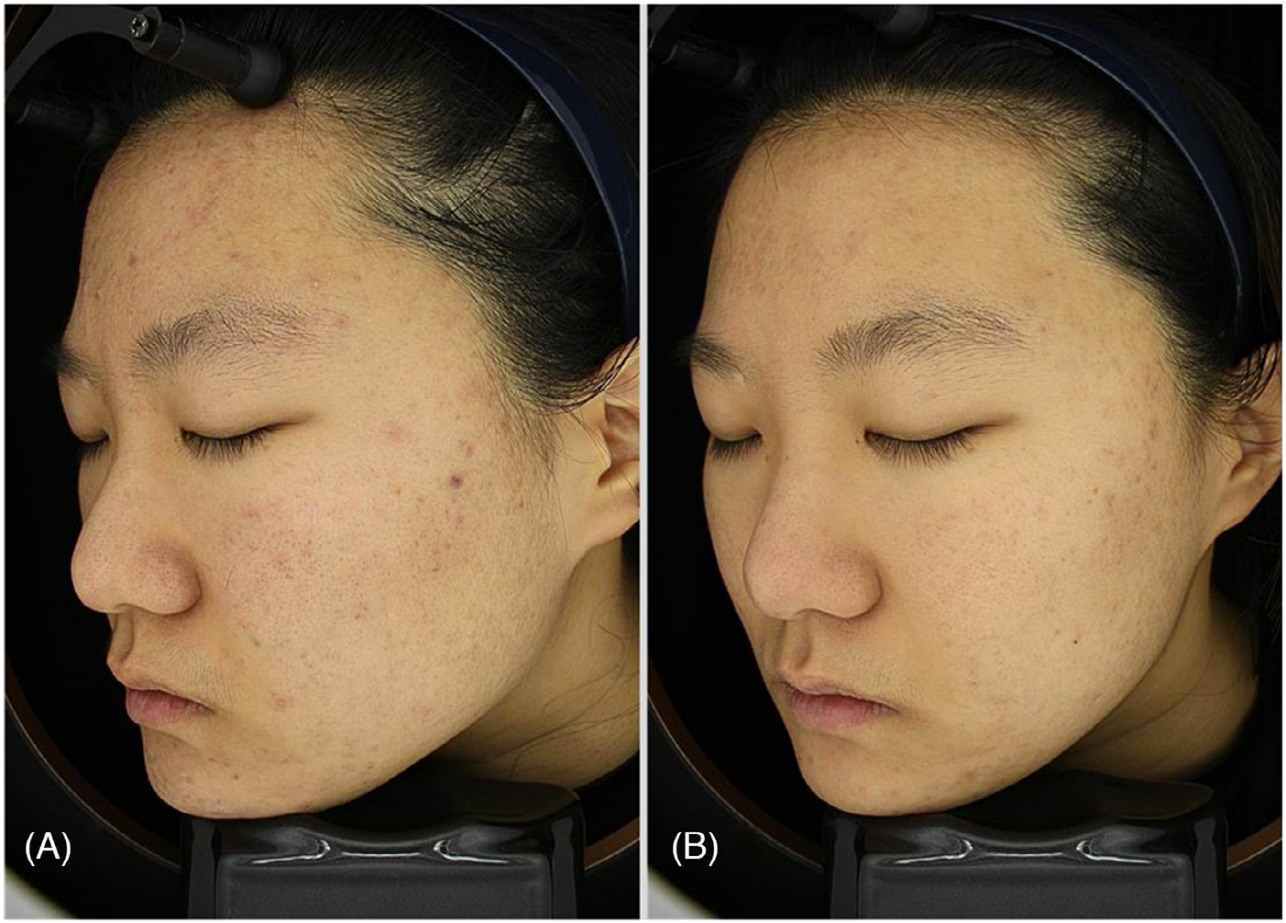
A 16‐year‐old patient came in had chief complaints of acne and had three treatment sessions with ethosome PTT (ETHOSOMEPTT, N‐Finders Co., Ltd, Korea) with Q1064 laser combination therapy. The image of before the treatment (A) and after the treatment (B).

When ethosome PTT generates instantaneous heat exceeding 60°C through the SPR effect, it can destroy surrounding sebaceous glands and promote collagen synthesis. This reaction also helps reduce inflammatory lesions. The penetrated metal nanoparticles are not absorbed into the body and are naturally excreted after approximately four weeks. Therefore, scheduling the procedure at intervals of 1–2 weeks allows the residual metal particles from the first ethosome PTT treatment to enhance the effectiveness of subsequent sessions.^12^, ^13^, ^14^, ^15^

As previously mentioned, ethosome PTT consists of a total of five layers, with an additional ethosome membrane on the innermost layer to encapsulate active ingredients. The whitening solution includes effective components for skin whitening such as ascorbic acid, niacinamide, tranexamic acid, ethyl ascorbyl ether, ascorbyl glucoside, and glutathione. tranexamic acid plays a role in blocking the activity of melanocyte cells responsible for melanin production.^16^, ^17^ Ascorbic acid, tranexamic acid, ethyl ascorbyl ether, and ascorbyl glucoside all function to inhibit the production of melanin pigment.^18^, ^19^ Niacinamide prevents the transfer of melanin pigment to the epidermis.^18^, ^20^ Glutathione, a powerful antioxidant, plays a role in preventing the oxidation of reactive oxygen species (ROS).^21^, ^22^, ^23^ The anti‐acne solution includes salicylic acid, levulinic acid, ascorbic acid, niacinamide, tranexamic acid, ethyl ascorbyl ether, and sodium hyaluronic acid. salicylic acid and levulinic acid play roles in alleviating acne inflammation.^24^ Niacinamide, tranexamic acid, and ethyl ascorbyl ether help prevent and treat post‐inflammatory hyperpigmentation associated with acne by inhibiting the production and transfer of melanin.^25^, ^26^, ^27^ Sodium hyaluronic acid performs a moisturizing function.

The use of ethosome PTT offers several significant advantages. It is a painless procedure with a shorter treatment time and no side effects compared to traditional PDT. Additionally, the SPR effect of the penetrated gold nanoparticles effectively destroys sebaceous glands without causing scars, regulates sebum secretion, promotes collagen synthesis, and enhances skin regeneration, thereby strengthening the skin barrier. One of the major benefits of ethosome PTT over traditional gold PTT is its ease and speed of penetration (Figure 2).

**FIGURE 2:**
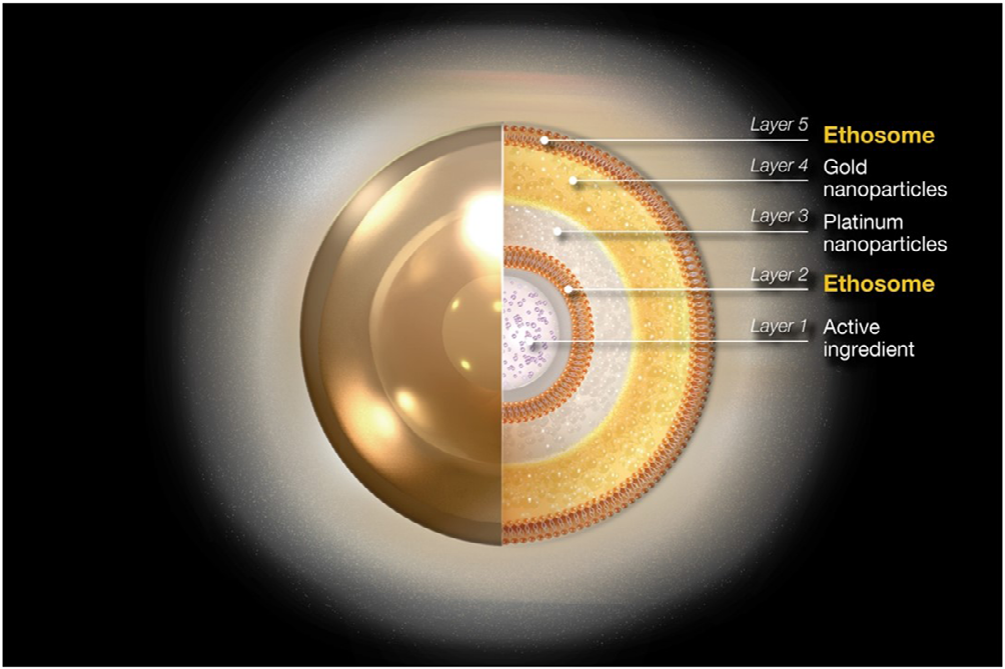
Ethosomes are the latest generation of liposomes, developed and proposed by professor E. Touitou and several other scientists in 2000. These advanced liposomes have been acknowledged in international academic circles. They possess unique electrical properties, characterized by a subtle electric charge. The image represents ETHOSOMEPTT (N‐Finders Co., Ltd, Korea).

Furthermore, the inclusion of active ingredients not found in previous products allows for the selection of solutions based on specific lesions, enhancing treatment efficiency. Ethosome PTT has clear advantages over both traditional PDT and gold PTT, providing efficient and effective treatment. While combined treatments are important in current therapeutic trends, and additional medication or procedures might be necessary in some cases, ethosome PTT alone possesses the characteristics of a combined treatment, offering substantial efficacy.

## CONFLICT OF INTEREST STATEMENT

The authors declared no potential conflicts of interest with respect to the research, authorship, and publication of this article. This study was conducted in compliance with the principles set forth in the Declaration of Helsinki.

## Data Availability

The data that support the findings of this study are available from the corresponding author upon reasonable request.
